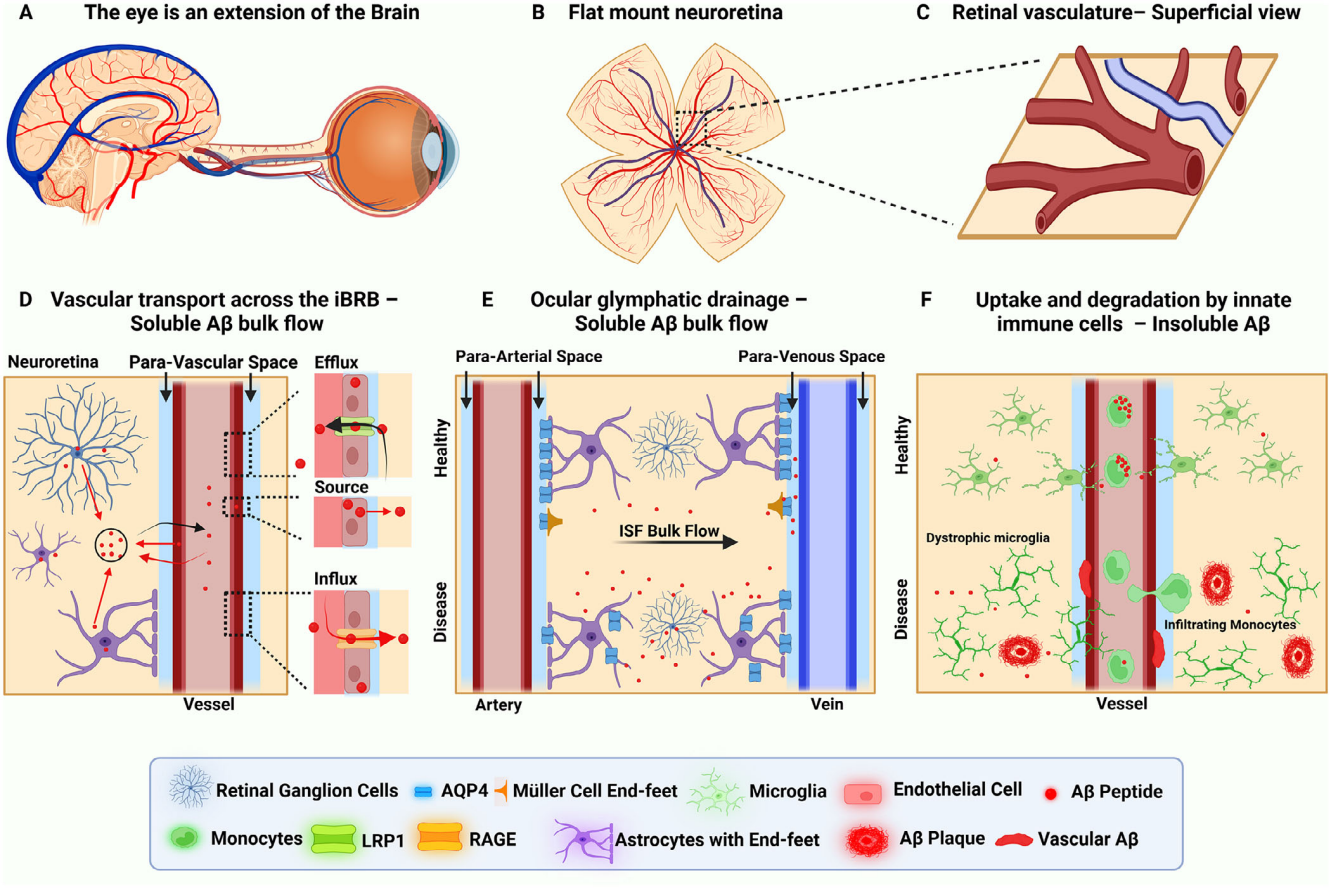# Impaired Aβ Clearance Mechanisms at the Inner Blood‐Retina Barrier in Alzheimer's Disease: Insights from Ex Vivo Retinal Imaging

**DOI:** 10.1002/alz70855_104480

**Published:** 2025-12-24

**Authors:** Printha Wijesinghe, Amir Hosseini, Matthew Campbell, Shivani Tejpal, Justin R Haynes, Jeanne Xi, Ian R MacKenzie, Veronica Hirsch‐Reinshagen, Ging‐Yuek Robin Hsiung, Benjamin Spiller, Brian E Wadzinski, Wellington Pham, Joanne A Matsubara

**Affiliations:** ^1^ The University of British Columbia, Vancouver, BC, Canada; ^2^ Vanderbilt University Medical Center, Nashville, TN, USA; ^3^ University of British Columbia, Vancouver, BC, Canada; ^4^ Djavad Mowafaghian Centre for Brain Health, Vancouver, BC, Canada

## Abstract

**Background:**

The imbalance between amyloid‐beta (Aβ) production and clearance is a key factor in the pathogenesis of sporadic Alzheimer's disease (AD). This study investigates impaired clearance mechanisms by examining interactions among neuronal and glial cells, retinal vasculature, and blood‐derived macrophages, particularly at the inner blood‐retina barrier (iBRB), a functional analog of the blood‐brain barrier (BBB), using wholemount neuroretinas and three‐dimensional ex vivo imaging. These interactions cannot be similarly studied in brain tissues due to its highly complex structure.

**Methods:**

Wholemount neuroretinas from human AD donors (*N* = 10, mean age ± SD: 76.8 ± 9.9 years; 6 males) and controls (*N* = 10, mean age: 72.5 ± 2.2 years; 5 males), as well as eye and brain cross‐sections from APP‐PS1 mice and controls (3 and 9 months, all females, N = 4 per group), were analyzed using three‐ and two‐dimensional ex vivo retinal imaging. Immunolabeling markers included Aβ1‐42 peptides (12F4), soluble Aβ1‐42 oligomers (SAβOs, NIR‐E3 nanobody), macroglia (GFAP, GS), microglia/macrophages (IBA1), water channels (AQP4), and retinal blood vessel endothelium (UEA‐1).

**Results:**

Human AD wholemount neuroretinas exhibited a significant increase in 12F4^+^ Aβ deposits (*p* < 0.0001) and IBA1^+^ disease‐associated microglia/macrophages (*p* = 0.0036), accompanied by reduced levels of macroglial markers GFAP (*p* = 0.0025), GS (*p* = 0.0015), and AQP4 (*p* = 0.0121), which are essential for glymphatic drainage, compared to age‐matched controls. Clearance of SAβOs by peripheral macrophage‐like monocytes through retinal blood vessels was also significantly diminished in AD retinas (*p* < 0.0001). In transgenic mouse retinal cross‐sections, increased GFAP, AQP4, and IBA1 levels, along with elevated APP/Aβ peptides, indicated gliosis and AQP4 dysregulation, contributing to impaired clearance systems compared to sibling controls.

**Conclusion:**

The imaging plane (wholemount vs. cross‐section) may influence outcomes in AD pathogenesis studies. Wholemount analysis revealed glymphatic clearance and microglial phagocytosis as compensatory mechanisms for mitigating Aβ accumulation, with peripheral macrophage‐like monocytes contributing to SAβO clearance in control neuroretinas. These mechanisms were largely disrupted in AD donors. Ex vivo 3D retinal imaging, applied here for the first time to study clearance at the iBRB, provides novel insights into retinal and BBB analog processes in AD.